# Supplementary material for: Beyond overconfidence: Embedding curiosity and humility for ethical medical AI
Source: PLOS Digit Health. 2026 Jan 5;5(1):e0001013. doi: 10.1371/journal.pdig.0001013 (PMC12768375; doi:10.1371/journal.pdig.0001013)
Supplement: Attachment — Submitted filename: response to reviewers.pdf [file pdig.0001013.s002.pdf]

Response to Reviewer Comments "Beyond Overconfidence: Embedding Curiosity and Humility for Ethical Medical AI"

Dear Editor and Reviewers,

We are grateful for the thoughtful and constructive feedback on our manuscript "Beyond Overconfidence: Embedding Curiosity and Humility for Ethical Medical AI." We have carefully addressed all reviewer comments, which have substantially improved the clarity, depth, and rigor of our work. Below is our detailed point-by-point response.

Sincerely, Sebastián Andrés Cajas Ordóñez, Rowell Castro, Leo Anthony Celi, Roben Delos Reyes, Justin Engelmann, Ari Ercole, Almog Hilel, Mahima Kaila, Leo Kinyera, Maximin Lange, Torleif Markussen Lunde, Mackenzie J Meni, Anna E Premo, and Jana Sedlakova

---

REVIEWER #1

Comment: Thank you for the opportunity to review this manuscript. I thoroughly enjoyed reading it. It is a thought-provoking and persuasive piece. The conclusions are well-supported by a range of literature and it provides essential insights for healthcare practitioners, technology specialists and healthcare system managers and policy makers. Well done!

Response: We sincerely thank you for your thoughtful engagement with our work and are gratified that you found the manuscript thought-provoking and well-supported. Your positive assessment strengthens our confidence that this framework will resonate with the diverse stakeholders you mention: practitioners, technologists, managers, and policymakers. We appreciate your generous feedback.

REVIEWER #2

Comment: This is a highly interesting and valuable paper that will contribute to improving the quality of future medical care. However, there are sections that require additional explanation and elaboration, so I would appreciate the authors' addressing these points.

Response: We thank you for this encouraging assessment and for your constructive suggestions, which have substantially strengthened the manuscript. Below we address each of your specific points:

Point 1: Connection between historical cases and curiosity/humility

Comment: In the Introduction, the authors mention forced castration and the Tuskegee syphilis study. Further explanation is needed regarding the connection between these two historical cases and the virtues of curiosity and humility, which are the central themes of this paper. These historical events cannot be explained solely by a lack of curiosity and humility; I believe that numerous other factors contributed to triggering these incidents. Since the

conclusion also references these events, relevant explanation should be added to one of these sections.

Response: Thank you for this crucial observation. You are absolutely correct that forced sterilizations and the Tuskegee study cannot be reduced to failures of curiosity and humility alone. These were multifactorial tragedies involving systemic racism, structural power imbalances, dehumanization, and deliberate ethical violations. We have revised both the Introduction and Conclusion to clarify that epistemic virtues function as necessary but not sufficient conditions for ethical medical practice. The new text explicitly acknowledges the broader social, political, and moral failures while demonstrating how the systematic absence of curiosity (failure to question foundational assumptions, explore alternative explanations) and humility (unwarranted certainty, refusal to acknowledge knowledge limits, absence of deference to affected communities) enabled these harms to persist unchecked. This framing positions our dual-reflective framework as one essential safeguard among many required for ethical AI.

Point 2: Virtues required of physicians using AI

Comment: This paper centers on discussing AI equipped with curiosity and humility, detailing how to endow AI with these two virtues. However, it lacks discussion on the virtues required of the physicians using such AI. To maximize patient care benefits, the paper should add discussion on what virtues are necessary for physicians using AI, and further, how to cultivate these necessary virtues in physicians.

Response: We thank you for identifying this important gap in our original manuscript. We have added substantive discussion in Section 5 ("What Does It Mean for AI to Make Us Better Humans?") that explicitly addresses physician virtues essential for productive engagement with dual-reflective AI systems: critical discernment (evaluating algorithmic recommendations with calibrated trust), reflexive awareness (monitoring one's own cognitive biases during AI interaction), and collaborative humility (treating AI as thinking partner rather than oracle). We emphasize that technical competence alone proves insufficient and argue that medical education must evolve to cultivate these epistemic virtues through case-based training with AI-discordant scenarios, simulation exercises revealing automation bias, and reflective practice protocols. This addition clarifies that our framework requires reciprocal virtue development in both AI systems and human clinicians.

Point 3: Author contributions

Comment: Finally, the current description of Author Contributions is extremely inadequate. It is necessary to clearly state how each of the fourteen authors contributed to the creation of this paper.

Response: Thank you for highlighting the need for more detailed author contributions, which we agree is of particular importance in manuscripts such as this with a large number of authors. We have expanded the Author Contributions section to provide specific details regarding each author's contributions across different phases of manuscript development. The revised contributions statement now clearly delineates: (1) conceptual development and early framework design, (2) initial manuscript drafting and writing, (3) critical revisions and conceptual refinement, and (4) final review and approval. Each author's specific role within

these phases is now explicitly stated, making clear that each author provided substantive intellectual contributions deserving of authorship.

REVIEWER #3

Comment: Thank you for your valuable article. It is suggested that the authors explain more about how the idea for this article was formed and which part of the article adds to the existing literature in the content.

Response: Thank you for this valuable suggestion. We have added explicit clarification in the Introduction regarding: (1) the intellectual genesis of this work, explaining how it extends our earlier Lancet publication by developing a comprehensive operational framework for implementing curiosity and humility in medical AI; and (2) three specific contributions to existing literature. First, we formally integrate curiosity and humility as complementary architectural principles grounded in both computational theory and cross-species cognitive science, moving beyond their treatment as separate technical or philosophical concerns. Second, we operationalize their synergistic implementation through context-sensitive activation frameworks (illustrated in Figure 1). Third, we demonstrate how these virtues must be cultivated across both algorithmic and human dimensions of sociotechnical systems, not merely embedded in code. This revised framing makes our intellectual trajectory and novel contributions substantially clearer for readers.

JOURNAL REQUIREMENTS

We have addressed all journal requirements as follows:

1. Manuscript source file: We have uploaded the manuscript as a .docx file.

2. Figure files: We have uploaded separate figure files in .tif format and removed figures from the manuscript file while keeping the legends.

3. Author Summary: We have added a 150-200 word Author Summary between the Abstract and Introduction that makes our findings accessible to a wide audience including both scientists and non-scientists.

4. Data Availability Statement: We have provided a complete Data Availability Statement in the submission form: "All data are in the manuscript and/or supporting information files."
